# Metal Surface Engineering for Extreme Sustenance of Jumping Droplet Condensation

**DOI:** 10.1021/acs.langmuir.3c02713

**Published:** 2023-12-29

**Authors:** Matteo Donati, Kartik Regulagadda, Cheuk Wing Edmond Lam, Athanasios Milionis, Chander Shekhar Sharma, Dimos Poulikakos

**Affiliations:** † Laboratory of Thermodynamics in Emerging Technologies, Department of Mechanical and Process Engineering, ETH Zurich, Sonneggstrasse 3, 8092 Zurich, Switzerland; ‡ Thermofluidics Research Laboratory, Department of Mechanical Engineering, Indian Institute of Technology Ropar, Rupnagar 140001, Punjab, India

## Abstract

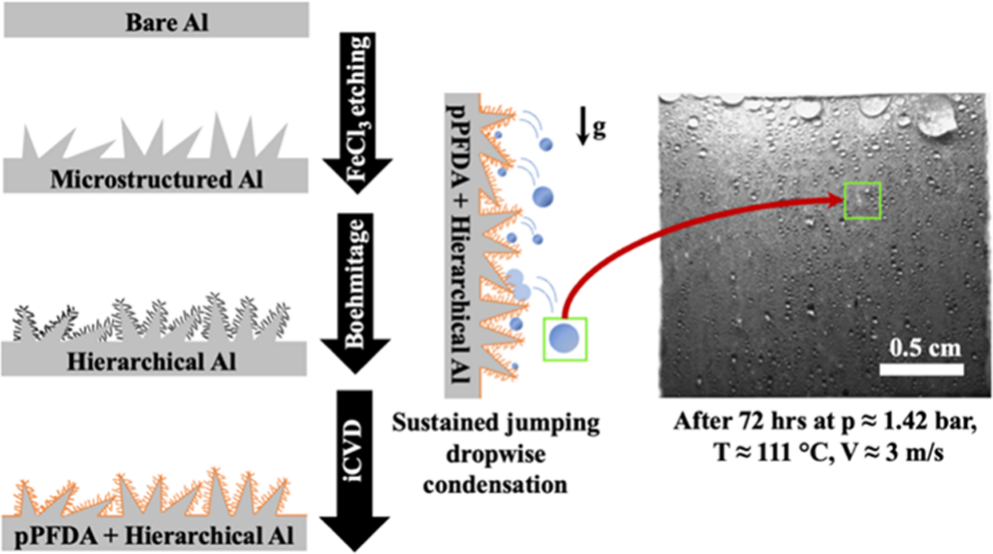

Water vapor condensation on metallic surfaces is critical to a broad range of applications, ranging from power generation to the chemical and pharmaceutical industries. Enhancing simultaneously the heat transfer efficiency, scalability, and durability of a condenser surface remains a persistent challenge. Coalescence-induced condensing droplet jumping is a capillarity-driven mechanism of self-ejection of microscopic condensate droplets from a surface. This mechanism is highly desired due to the fact that it continuously frees up the surface for new condensate to form directly on the surface, enhancing heat transfer without requiring the presence of the gravitational field. However, this condensate ejection mechanism typically requires the fabrication of surface nanotextures coated by an ultrathin (<10 nm) conformal hydrophobic coating (hydrophobic self-assembled monolayers such as silanes), which results in poor durability. Here, we present a scalable approach for the fabrication of a hierarchically structured superhydrophobic surface on aluminum substrates, which is able to withstand adverse conditions characterized by condensation of superheated steam shear flow at pressure and temperature up to ≈1.42 bar and ≈111 °C, respectively, and velocities in the range ≈3–9 m/s. The synergetic function of micro- and nanotextures, combined with a chemically grafted, robust ultrathin (≈4.0 nm) poly-1*H*,1*H*,2*H*,2*H*-perfluorodecyl acrylate (pPFDA) coating, which is 1 order of magnitude thinner than the current state of the art, allows the sustenance of long-term coalescence-induced condensate jumping drop condensation for at least 72 h. This yields unprecedented, up to an order of magnitude higher heat transfer coefficients compared to filmwise condensation under the same conditions and significantly outperforms the current state of the art in terms of both durability and performance establishing a new milestone.

## Introduction

Heterogeneous condensation of water on a solid surface is ubiquitous in nature, e.g., when dew is collected overnight on all kinds of natural surfaces, from grass^1^ to spider silks,^2^ as well as in engineering applications such as energy conversion cycles of power plants,^3^ water collection (dew) from atmospheric air,^4^ separation technologies,^5^ and electronics cooling.^6^ Water deposition on surfaces through condensation can be a result of either phase transition or water supersaturation of the surrounding air with respect to the surface temperature and can be categorized into two distinct modes, namely, filmwise condensation (FWC) and dropwise condensation (DWC).

Typically, on a hydrophilic surface, we observe FWC due to condensate accumulation leading to a uniform, thermally insulating water film significantly hindering the heat transfer through the surface.^7^ DWC, typical of hydrophobic surfaces, is characterized by a regular condensate shedding in the form of distinct droplets via, e.g., gravitational forces or an external shear flow.^3,8−11^ This leads to an enhancement in heat transfer coefficient (HTC) up to an order of magnitude compared to FWC.^3^ Therefore, DWC is more desirable for efficient heat and mass transport applications.^3^

Interestingly, on some nanostructured superhydrophobic surfaces, DWC heat transfer can be further enhanced due to a droplet removal mechanism independent of gravity, known as coalescence-induced droplet jumping.^12−14^ This physical capillary-driven phenomenon is characterized by partial conversion of excess surface energy into kinetic energy after coalescence of two or more droplets^15−19^ and allows the ejection of significantly smaller droplets compared to standard DWC, down to 500 nm in diameter.^20^ This gravity-independent mode of condensate departure due to coalescence^15,18^ is often termed as jumping dropwise condensation (JDWC). It has yielded up to ≈2× higher HTC compared to DWC.^12^ However, sustaining JDWC for prolonged periods is still an ongoing challenge due to the requirement of robust nanotextures in combination with a strong ultrathin (<10 nm) conformal coating (an essential characteristic to minimize the overall thermal resistance).^12^

Since the discovery of JDWC, nanostructured superhydrophobic surfaces have garnered significant interest. However, they usually lack long-term durability during condensation. The fragility of nanostructures and the usual weakness of the popular ultrathin hydrophobic conformal coatings make them susceptible to damage, induced by shear flow of the vapor or the condensate itself.^21−25^ Furthermore, nanostructured superhydrophobic surfaces are prone to flooding at high supersaturations due to the excessive rate of nucleation and the decrease in the critical nucleation diameter.^26−28^

It is well-known that the presence of microtextures underneath nanostructures offers several advantages compared with solely nanostructured surfaces. First, microstructures can enhance the surface mechanical durability and resilience against e.g., shear stresses. In fact, while the exposed top nanostructures can be easily removed, the ones within the microtopography are protected by the microtextures that act as a protective shield.^21,22,29^ Consequently, hierarchically structured surfaces (HSS) yield more robust superhydrophobicity^30,31^ and can sustain JDWC for longer times.^23,24^ Second, HSS can exhibit higher HTC improvement due to the larger effective surface area available for condensation and better droplet mobility.^24,32,33^ Last, the deformation of condensing microdroplets growing from within the microcavities induces an internal Laplace pressure imbalance. This translates into a net out-of-plane force facilitating the depinning of droplets in a partial or complete nano-Wenzel state to a micro-Cassie state, eventually leading to their ejection from the surface.^8,23,32,33^ Despite all this, JDWC on HSS has only been addressed under mild conditions,^24,25,32−38^ while their critical to possible applications durability performance under condensation exposure remains a challenge.

With respect to industrial condenser applications, metals and in particular aluminum (Al), due to its lightweight and high thermal conductivity, up to 237 W/(K·m),^39^ are highly desirable.^40,41^ Unfortunately, aluminum results in FWC due to the formation of hydrophilic boehmite upon exposure to steam. Its surface is usually hydrophobized by means of conformal ultrathin organic coatings mostly consisting of silanes, deposited with dip-coating,^42,43^ spin-coating,^44^ or chemical vapor deposition (CVD).^45^

Initiated chemical vapor deposition (iCVD) is a scalable method for the application of robust polymer coatings, like, for example, poly-1*H*,1*H*,2*H*,2*H*-perfluorodecyl acrylate (pPFDA).^46^ In this technique, pPFDA, a material with ultralow surface energy of ≈7 mJ·m^–2^,^47^ is chemically grafted to a substrate with well-controlled thickness (≈30–300 nm),^9,46,48−50^ which is markedly stronger compared to silanes.^9,46,49^ Its use for the hydrophobization of plain metallic surfaces successfully promoting DWC has already been shown in the literature.^9,46,49^ However, in the case of nanostructured surfaces promoting JDWC, the coating thickness must be further lowered to ensure conformality. We achieved this by optimizing the iCVD coating process, being able to reduce the pPFDA thickness by one order of magnitude compared to the current state of the art.^9,46,48−50^

Here, we investigated the condensation heat transfer performance to superheated steam shear flow at ≈111 °C, ≈1.42 bar, and velocity ≈3–9 m/s of two different types of silane monolayers (trichloro-1*H*,1*H*,2*H*,2*H*-perfluorodecylsilane, FDTS and 1*H*,1*H*,2*H*,2*H*-perfluorodecyltriethoxysilane, PFDTS), and an ultrathin (≈4.0 nm thick) pPFDA-grafted polymer, deposited onto aluminum substrates. The silanes and pPFDA were applied via CVD and iCVD, respectively. We showed that only the pPFDA surface can survive the harsh environment of the experiments. We thus applied this coating to a hierarchically structured aluminum substrate (H-pPFDA). The H-pPFDA exhibited sustained JDWC accompanied by HTC improvement of up to ≈9.6× compared to FWC. Additionally, we tested the H-pPFDA surface for durability by means of an accelerated aging test consisting of continuous condensation under the same aforementioned challenging conditions by keeping the steam velocity at ≈3 m/s. During the entire experiment, which we terminated after 72 h due to practical limitations in performing it, the H-pPFDA surface yielded JDWC combined with >8.3× higher HTC compared to FWC, without any significant sign of degradation. We finally compared the H-pPFDA surface durability in terms of temporal droplet departure diameter evolution during a similar endurance test with the state-of-the-art robust poly(tetrafluoroethylene)/carbon nanofibers coating^12^ (PTFE/CNF). The H-pPFDA surface outperformed the PTFE/CNF surface by exhibiting JDWC for at least 7× longer time, with no changes in droplet departure diameter, significantly surpassing the state of the art and thus establishing a new performance milestone.

## Experimental Results

### Surface Topography and Wettability Characterization

We fabricated the surface topographies used in this study on aluminum substrates. The flat surfaces were functionalized with FDTS and PFDTS via CVD. More details about CVD can be found in the Methods Section. Surface microstructuring was achieved by means of dislocation-selective etching using iron(III) chloride,^23^ while the nanostructures were fabricated via the boehmitage process in hot water.^23,51,52^

**Figure 1:**
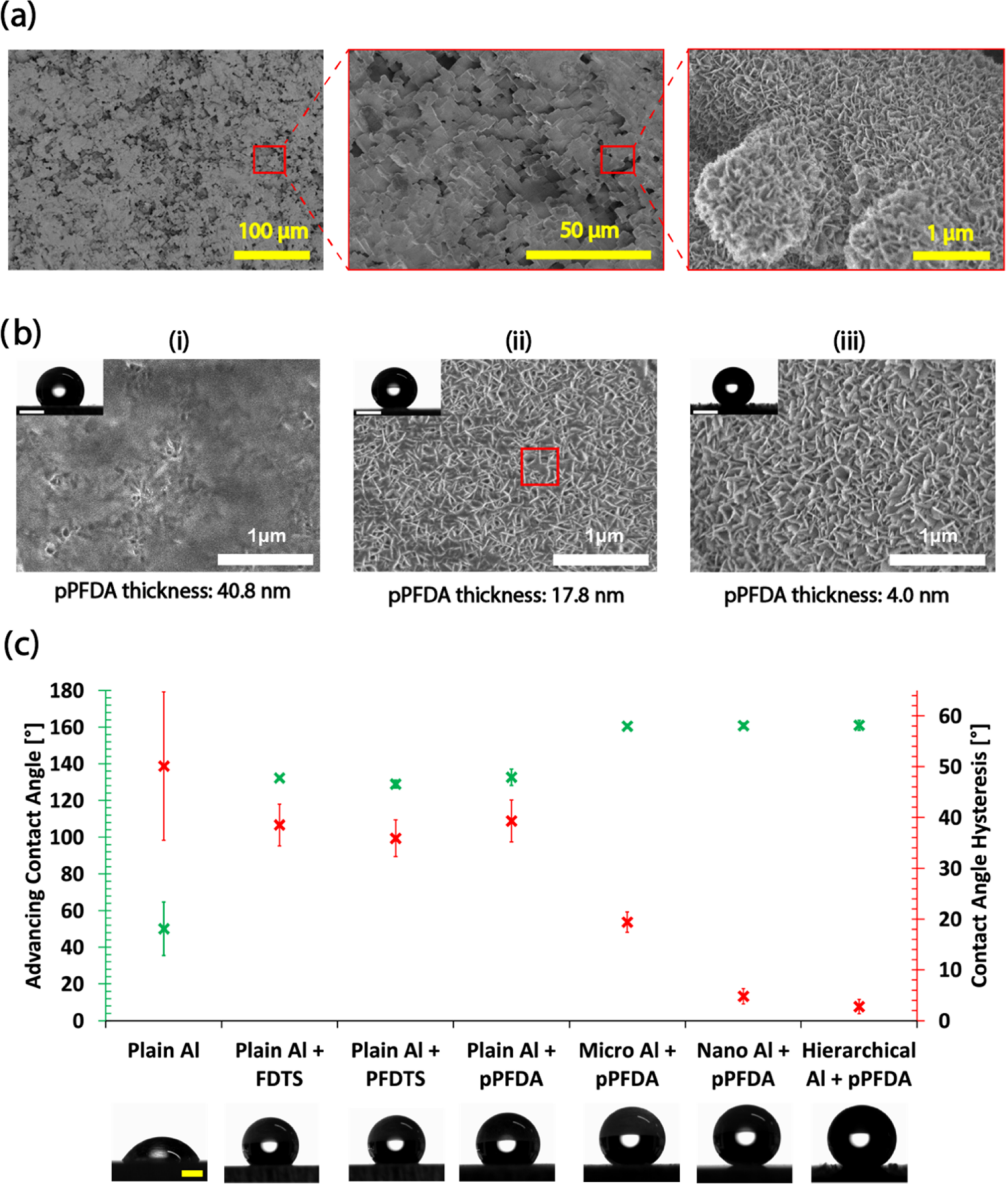
(a) Surface micro- and nanotopography of the superhydrophobic H-pPFDA surface. (b) SEM micrographs of nanostructured aluminum coated with different pPFDA thicknesses. Insets show a sessile water droplet with a volume of 5 μL on the different surfaces. The red square in panel (ii) highlights one of the spaces between nanostructures flooded with pPFDA. Inset scale bars: 2 mm. (c) Advancing contact angle (ACA) and contact angle hysteresis (CAH) of the surfaces included in this study. Insets show a sessile water droplet with a volume of 5 μL on the different surfaces. Scale bar: 1 mm.

Figure 1a displays the topography of H-pPFDA at micro- and nanoscales. The topography is characterized by random microstructures with re-entrant cavities as observed in Sharma et al.,^23^ combined with highly dense nanowalls consisting of boehmite.^23^ This structural architecture is beneficial for efficient and enhanced condensate removal.^23^

As far as the pPFDA coating is concerned, we applied it with iCVD.^9,46^ Based on the work of Walker et al.^53^ and Mitridis,^54^ we further optimized the pPFDA deposition process, mainly in terms of deposition time, targeting controlled deposition rate, and process reproducibility. The objective was to minimize the thickness and ensure the coating conformality on the nanotextures. This aspect was essential to promote JDWC. The current state of the art reports pPFDA thicknesses down to ≈30 nm,^50^ which would lead to a loss in superhydrophobicity when applied to a nanostructured surface. An overview of the different pPFDA coatings presented in the literature is summarized in Supporting Information S1. We therefore coated nanostructured Al with three different pPFDA thicknesses, ranging from ≈40.8 nm down to ≈4.0 nm, where the thickness was varied by changing the deposition time. We characterized each surface in terms of wettability. Figure 1b shows the nanoscale morphology of the surfaces for different pPFDA thicknesses. By applying a ≈40.8 nm thick pPFDA layer, the nanostructures were almost completely covered (panel (i)). The effect of nanotextures on hydrophobicity was therefore suppressed. In this case, the advancing contact angle (ACA) and the contact angle hysteresis (CAH) were 138.1 ± 2.1 and 22.9 ± 3.8°, respectively. By reducing the pPFDA thickness down to ≈17.8 nm, the space within the nanostructures was partially filled with the polymer (panel (ii)). This led to an ACA of 161.6 ± 2.1° combined with a CAH of 30.9 ± 1.6°. Finally, a pPFDA thickness of ≈4.0 nm (1 order of magnitude lower compared to the current state of the art) was completely conformal to the nanostructures leading to superhydrophobicity with low water adhesion (panel (iii)). Here, the ACA and CAH were 161.02 ± 1.4 and 2.8 ± 1.4°, respectively. We therefore selected ≈4.0 nm as the optimal thickness for our work. The procedure of the pPFDA coating thickness measurement is described in Supporting Information S2, and further details on its deposition via iCVD can be found in the Methods Section.

Figure 1c summarizes the ACA and the CAH of all of the surfaces included in this study. All of the values reported were determined based on the average of five measurements taken at random locations on the surfaces. Plain Al, the reference surface for FWC, is hydrophilic. Its ACA and CAH have the same value (50.1 ± 14.6°) since the receding contact angle is ≈0°. Plain Al coated with FDTS, PFDTS, and pPFDA are hydrophobic having similar ACA, i.e., 132 ± 1.1, 128.9 ± 2.1, and 132.6 ± 4.5°, respectively. The corresponding CAH values are 38.5 ± 4.1, 35.9 ± 3.6, and 39.3 ± 4.1°, respectively. Regarding the pPFDA-coated structured surfaces, the one with only microtextures has an ACA of 160.5 ± 1.2° and CAH of 19.4 ± 2°. The nanostructured-only and the H-pPFDA (i.e., hierarchical) surfaces have an ACA of 160.8 ± 1.5 and 161.02 ± 2.7° accompanied by CAH of 4.8 ± 1.5 and 2.7 ± 1.4°, respectively. Thus, all of the structured pPFDA-coated surfaces exhibit superhydrophobicity. Compared to the microstructured-only surface, the CAH on the H-pPFDA and the only nanostructured surfaces is significantly lower due to the reduced contact area between the droplet and substrate leading to lower adhesion forces.^55,56^

### Condensation Heat Transfer Performance Characterization

We evaluated all of the surfaces in terms of condensation heat transfer efficiency under extreme conditions in a custom-built condensation setup. The harsh environment consisted of superheated steam shear flow at ≈1.42 bar pressure and 111 ± 0.3 °C temperature, flowing vertically downward, parallel to gravity.^8,9,12,57^ The surfaces were tested at steam velocities of ≈3 and ≈9 m/s. Detailed information on the experimental setup and data acquisition can be found in Supporting Information S3.

**Figure 2:**
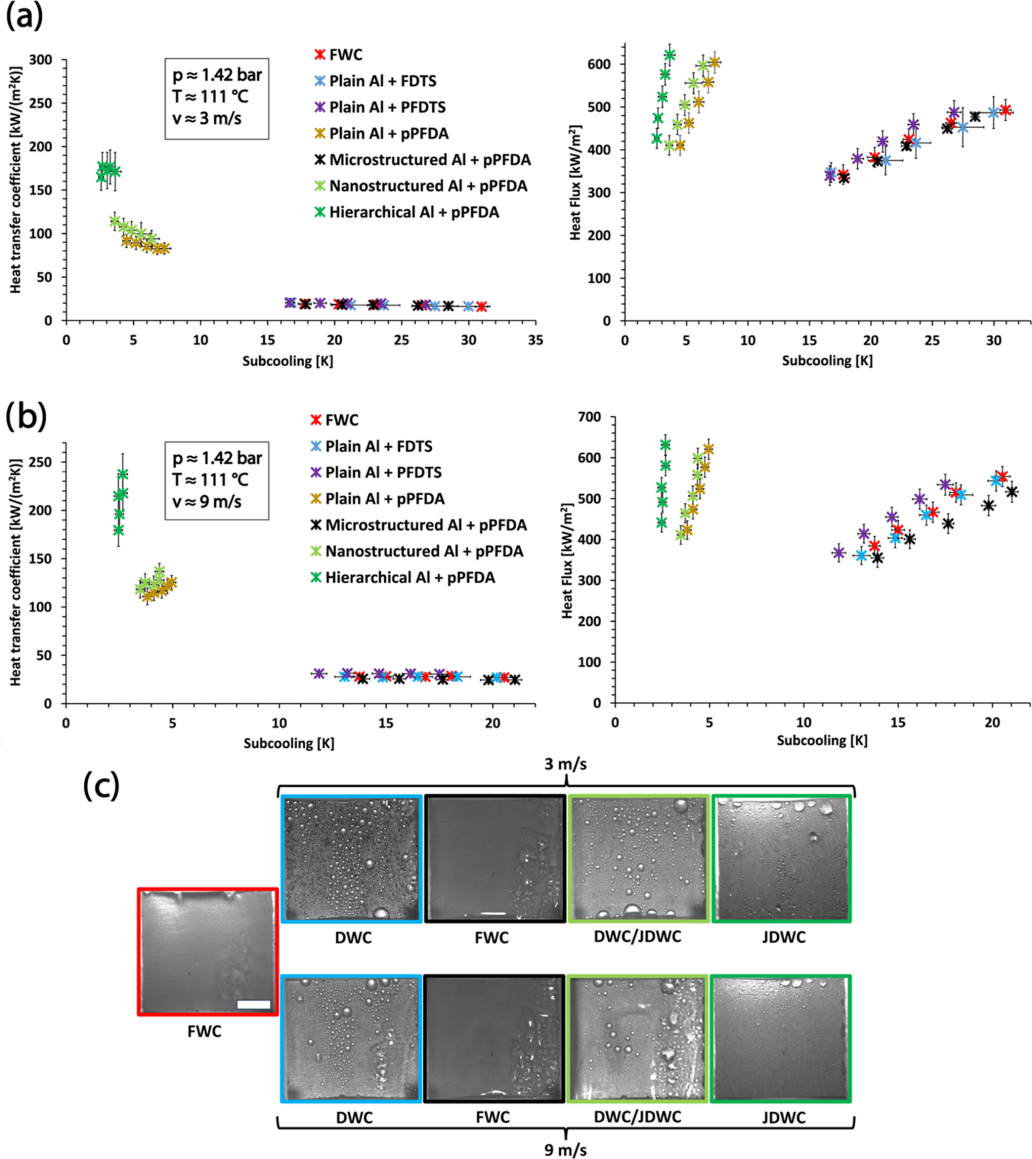
Heat transfer coefficient (left graphs) and heat flux (right graphs) for all of the substrates during condensation of superheated shear steam flow at ≈1.42 bar, ≈111 °C, and velocity of (a) 3 and (b) 9 m/s. The H-pPFDA surface has the highest HTC at both steam velocities owing to the synergy of micro- and nanotextures leading to high condensate removal. (c) Snapshots displaying the condensation mode on the different surfaces at 3 and 9 m/s. Scale bar in panel (c): 4 mm.

Figure 2a,b shows HTC and heat flux data of all of the surfaces at 3 and 9 m/s, respectively, as a function of the subcooling Δ*T* = *T* – *T*_s_, where *T* is the steam temperature and *T*_s_ is the surface temperature of the substrate. In general, the improvement in HTC for a surface showing DWC or JDWC compared to FWC is lower at 9 m/s. The increased steam velocity enhances nucleation and condensation rate.^58^ At the same time, the larger advective forces reduce the droplet departure diameter observed on the surface showing DWC or JDWC and the film thickness on the hydrophilic substrate in FWC.^57^ These factors enhance the HTC in all of the cases. The combination of the aforementioned effects on the surfaces displaying DWC or JDWC cannot compensate the HTC enhancement of FWC, leading to a consequent fall in condensation heat transfer performance improvement.^8,9,12,57^ For FDTS- and PFDTS-coated surfaces, the HTC values were in the same range as those of reference plain Al (i.e., FWC). Despite showing DWC initially, these surfaces failed, i.e., the hydrophobic chemical groups are removed and FWC is observed after ≈2 min of condensation exposure.

This can be explained by the fact that the silicon of the silane molecules form oxane bonds (Si–O) with the substrate, which are not hydrolytically stable and can easily be broken by the steam,^59,60^ starting from inherent defects that can be easily reached by the vapor.^61^ pPFDA is chemically grafted with the aluminum by means of the same chemical bonds. In this case, however, the coating is more stable when exposed to condensation compared to FDTS and PFDTS due to the presence of densely packed fluorine hydrophobic chains improving the protection of inherent surface defects.^62^ More details on this can be found in Supporting Information S4.

The flat pPFDA-coated surface manifested DWC with ≈4.8× and ≈4.2× higher HTC compared to FWC, at 3 and 9 m/s steam velocities, respectively. For this reason, we considered further only the pPFDA coating (rather than the silanes) for the textured surfaces used in the study. The pPFDA surface with only microstructures, although superhydrophobic, led to FWC. This is due to surface flooding (not coating removal) which originated from the incapability of the microstructures to eject the condensate owing to high nucleation and condensation rates;^14^ in fact, the wettability before and after the condensation heat transfer experiment for this surface remained similar (see Supporting Information S5). As a consequence, the HTC was similar to that measured for the FWC reference surface of plain Al. The nanostructured-only superhydrophobic surface yielded DWC due to partial flooding, mainly characterized by droplets remaining in the Wenzel state, which can still be removed via gravitational forces and advection, with a HTC ≈5.8× and ≈4.5× higher than FWC at steam velocities of 3 and 9 m/s, respectively.

Finally, the H-pPFDA surface resulted in JDWC owing to the synergy of micro- and nanotextures, leading to superior droplet mobility and condensate removal rate compared to all of the other pPFDA-coated surfaces. At the same subcooling (≈3.5 K), the nanostructured-only surface was partially flooded while no flooding was observed on the H-pPFDA surface (see condensation snapshots in Supporting Information S6). This is due to the absence of microstructures; microdroplets that are in a partial or total Wenzel state can only transition to Cassie–Baxter by coalescence with other droplets in their neighborhood. On the other hand, as extensively studied by Sharma et al.^23^ on a surface with similar structures, microdroplet transition to a nano-Cassie state on H-pPFDA is facilitated by the presence of an additional condensate removal mechanism characterized by the deformation of droplets growing within the microtextures and having sizes of the same order of magnitude as the microcavities. This induces an internal Laplace pressure imbalance, resulting in a net force pushing the droplet out of the cavity. This can aid the transition from a micro-Wenzel to micro-Cassie state, and eventually the droplet can leave the surface by coalescence-induced jumping. The latter can occur with or without further coalescence with droplets that nucleated on the top of the microtextures enhancing condensate removal.^8,23,24,32^ Compared to FWC, this translated into a significantly higher HTC of ≈9.6× and ≈7.5× at 3 and 9 m/s, respectively. The H-pPFDA was therefore the best performing surface of this study. During the full duration of the condensation heat transfer characterization experiment (≈3 to 4 h), all of the pPFDA-coated surfaces did not show any sign of degradation and exhibited the same condensation dynamics. The snapshots showing the condensation modes on these surfaces at 3 and 9 m/s are displayed in Figure 2c. The condensation dynamics on all of the aforementioned surfaces at steam velocity ≈3 m/s can be seen in Supporting Video S1.

Our experimental condensation setup is specifically designed to test the limits of the surface coatings by exposing them to adverse conditions, which are significantly harsher (in terms of vapor temperature and pressure) compared to the ones observed in conventional industrial condensers. These usually operate with saturated steam at pressures in the range of tens of millibars and temperatures close to ambient.^9,12,57,63^ Moreover, the narrow channel through which steam flows in our setup additionally exposes the test surfaces to extra shear stresses (e.g., 65 mPa at 3 m/s),^12^ accelerating their degradation.

For the sake of completeness, since H-pPFDA showed the best condensation heat transfer performance, we additionally tested it in a second condensation setup able to mimic the conditions of conventional industrial condensers. During exposure to condensation of saturated steam at ≈30 mbar, the surface yielded a ≈ 2.9× higher HTC compared to FWC. More details on this can be found in Supporting Information S7.

### Extended Durability Test

Since the H-pPFDA surface displayed exceptional condensation heat transfer performance, we tested its durability by means of an accelerated aging test conducted in the high-pressure experimental setup. During the entire experiment, the conditions of superheated steam were maintained at ≈111 °C, ≈1.42 bar, and shear velocity of ≈3 m/s. The experiment consisted of cycles with 6–9 h of condensation exposure for 9 consecutive days for a total of 72 h. After each cycle, the setup was shut down and restarted again the next day. During the restart, before steam generation in each cycle, the H-pPFDA surface was also exposed to liquid water shear flow as the fluid slowly transitioned to the vapor phase upon continuous heating fluid in the start-up of our setup,^8^ making this test even more extreme due to its ≈17× higher dynamic viscosity compared to steam.^8^ We monitored the transient condensation dynamics during the test by means of high-speed imaging as well as the HTC evolution.

**Figure 3:**
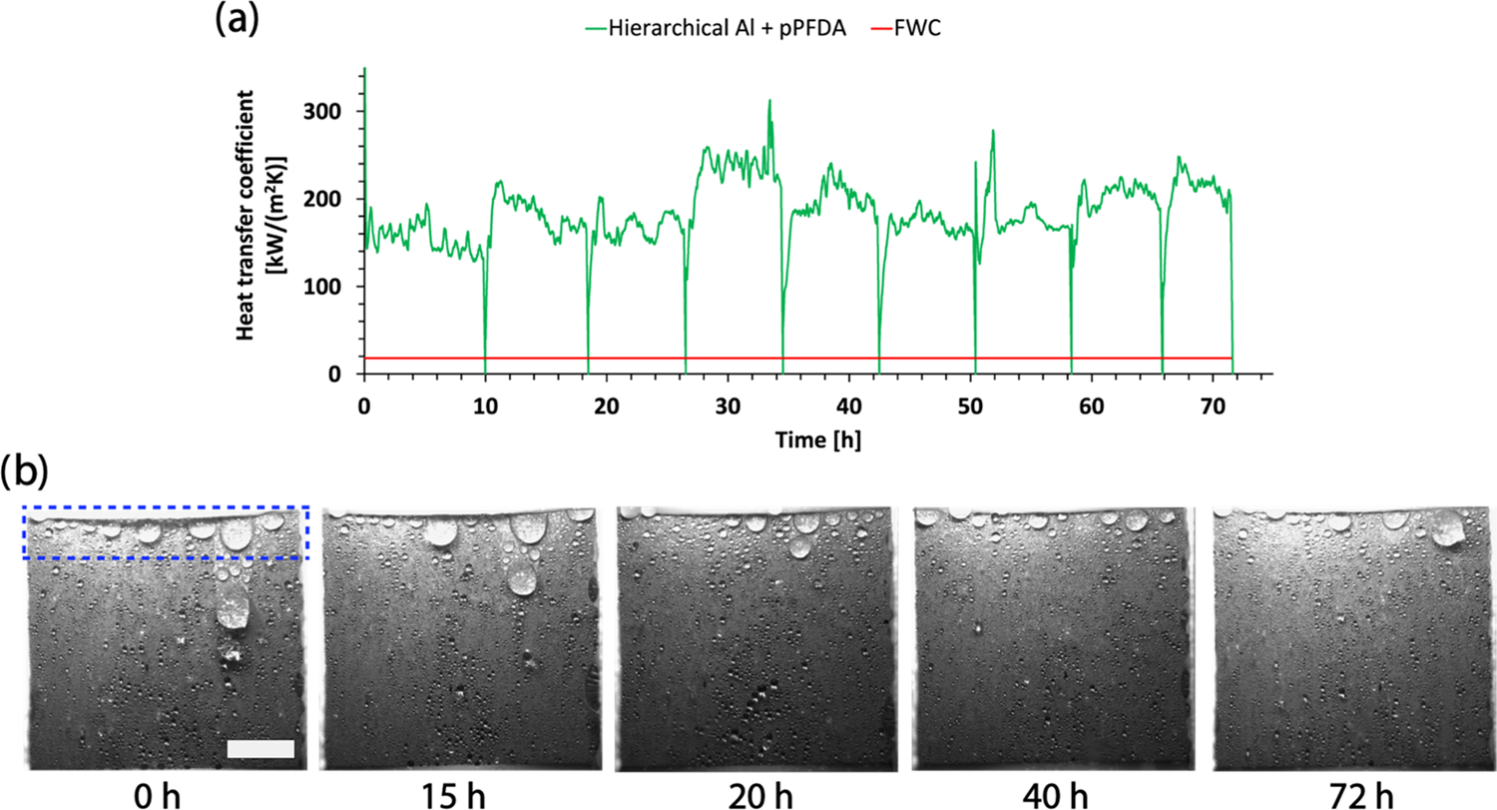
(a) HTC vs time of the H-pPFDA surface during the accelerated durability test. Between each steam exposure cycle, there is an obvious HTC drop. Red line represents the HTC on the reference plain Al substrate which displays FWC. (b) Snapshots acquired via high-speed imaging showing the condensation behavior on the H-pPFDA surface over time during the same test in panel (a). The larger droplets visible on the top surface section on the panels in (b) are due to edge effects from the finite sample size and have no relation to the surface durability. These are highlighted by the blue dashed rectangle on the snapshot at 0 h. Scale bar in panel (b): 5 mm.

Figure 3 shows the temporal HTC variation and condensation behavior, respectively. The periodic dips in HTC correspond to the steam shut down at the end of each day of experiment. The H-pPFDA surface displayed an exceptional durability characterized by HTC > 8.3× higher compared to FWC (red line in Figure 3a) throughout the full experiment duration. Supporting Video S2 displays the condensation dynamics, while Figure 3b displays snapshots, at different times during the durability test. It can be clearly observed that JDWC is sustained at all times without any degradation signs. Important to mention is that the larger droplets visible on the top surface section are due to edge effects from the finite size of our test samples and do not represent any failure of the surface durability at these locations. The aforementioned result is also supported by contact angle measurements, as well as micro- and nanotopography analysis via scanning electron microscopy performed on the H-pPFDA surface after the durability test. The micro- and nanostructure morphologies were unchanged (see Supporting Information S8), while the wettability, characterized by ACA and CAH of 161.8 ± 2.8 and 11.8 ± 6.1°, respectively, remained nearly constant compared to a fresh surface. We point out that the presented durability characterization in Figure 3 is a clear underestimation of the actual durability of our surface because the experiment was terminated well before surface degradation starts. This test is very time-consuming and requires continuous setup monitoring leading to practical personnel challenges in performing the experiment even further, leading to the decision to terminate it after 72 h of performance and nine consecutive days of running it.

### Comparison with the State of the Art

We compared the H-pPFDA durability with a state-of-the-art surface tested in a similar accelerated durability experiment conducted under the same conditions. This surface consists of a superhydrophobic nanocomposite of poly(tetrafluoroethylene) and carbon nanofibers (PTFE/CNFs).^12^ It is the only surface known to us and available in the literature that showed JDWC when exposed to a similar level of adverse conditions during condensation. Furthermore, when exposed to high-pressure steam flow condensation, at the vapor velocity of 3 m/s, this shows HTCs comparable with the ones observed on the H-pPFDA surface, while at 9 m/s, it performs slightly better (see Supporting Information S9).

**Figure 4:**
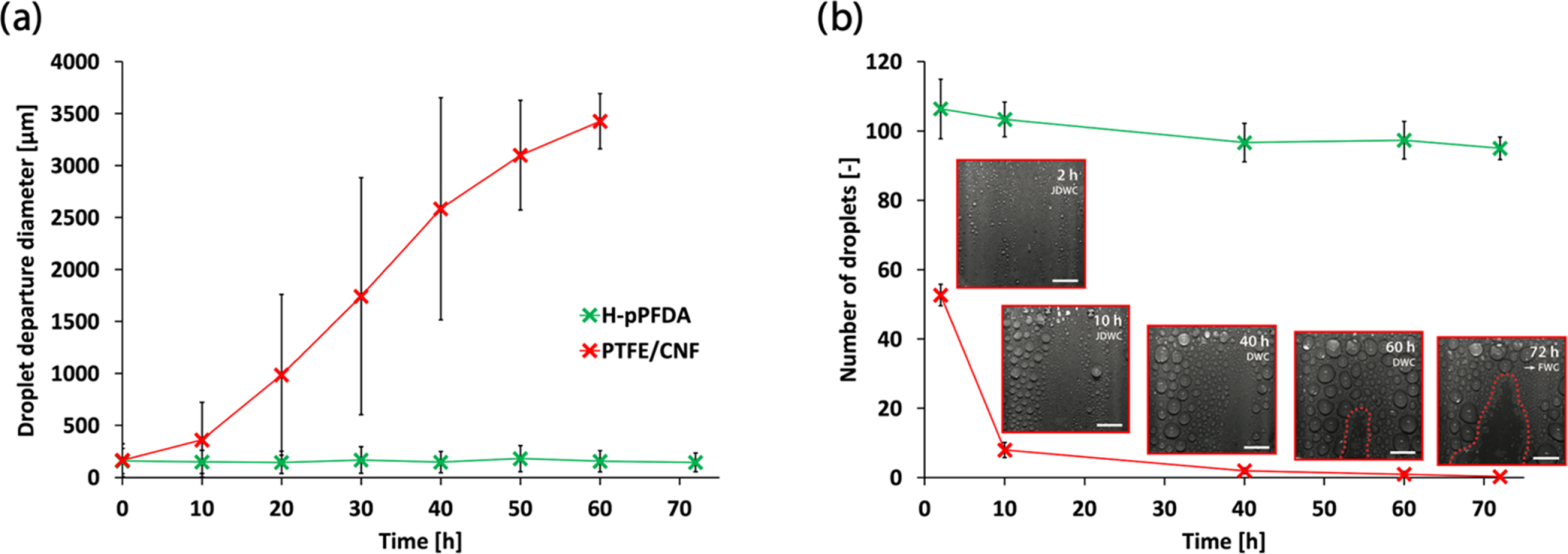
Temporal evolution of (a) mean droplet departure diameter (MDD) and (b) number of instantaneous ejected droplets (IED) during similar durability tests performed on H-pPFDA and PTFE/CNF surfaces. The red dashed lines on the snapshots in panel (b) highlight FWC regions. Snapshots in panel (b) are adopted from Donati et al.^12^ Scale bars in panel (b): 4 mm.

Figure 4a,b displays the temporal evolution of the mean droplet departure diameter (MDD) and number of instantaneous ejected droplets (IED), respectively, at different selected time instances during the accelerated durability test (more details on data collection and evaluation can be found in the Methods Section). The H-pPFDA surface produced JDWC and an unchanged MDD in the range ≈145–180 μm throughout the full experiment. On the other hand, the PTFE/CNF surface, after an initial MDD of ≈167 μm, had a constantly increasing MDD with time, reaching a size of ≈3.4 mm after 60 h.

As far as the IED is concerned, we considered only the data starting from 2 h of experiment due to obstructed view on the PTFE/CNF surface caused by fogging of the inner window side of our setup at the beginning of the experiment.^12^ For both surfaces, the area exposed to condensation and considered for this measurement was the same (400 mm^2^). H-pPFDA surface resulted in a constant number of IED (≈100 for each instant of time) during the whole experiment. However, the PTFE/CNF surface, starting from an IED number of ≈50, degraded significantly leading to IED number of <1 after 72 h. The increase in MDD and the reduction in IED number observed on the PTFE/CNF surface is also reflected in the condensate removal mechanism transitions. This surface could sustain JDWC for ≈10 h only, followed by ≈50 additional hours of DWC as the prevalent mode, and subsequent transition to FWC after ≈72 h caused by coating failure.

Summarizing, despite the state-of-the-art PTFE/CNF and H-pPFDA surfaces showing similar initial HTC’s when exposed to high-pressure steam flow condensation, the results presented in this section clearly demonstrate superior durability of the latter.

## Conclusions

We have demonstrated the remarkable condensation heat transfer performance as well as the long-term durability of a superhydrophobic hierarchically structured aluminum surface coated with a ≈4.0 nm thick pPFDA layer deposited by means of iCVD. Under extreme conditions, the surface exhibited a maximum HTC ≈9.6× higher compared to FWC. Furthermore, it was able to sustain JDWC without showing any clear signs of degradation for at least 72 h in a harsh environment, characterized by superheated shear steam flow at ≈1.42 bar, ≈111 °C, and with a velocity of ≈3 m/s. This condensation mode was sustained for at least 7.2× longer time compared to the control state-of-the-art PTFE/CNF surface^12^ tested under the same adverse conditions. Considering the exceptional condensation heat transfer performance, durability under operation, and scalability of the H-pPFDA surface fabrication, our work can find significant practical utility, enhancing the efficiency of condensation heat transfer systems over a broad palette of applications.

## Methods

### Substrate Cleaning

Flat 99.5% aluminum substrates (EN AW-1050A, Lasercut AG) were cleaned by sonicating in acetone, isopropanol, and deionized water for 10 min each.

### Fabrication of Microstructures

The clean aluminum substrates were first sonicated in ≈0.25 M sodium hydroxide for at least 10 min. The substrates were then rinsed immediately with deionized water and dried with nitrogen. For microstructure etching,^23^ iron(III) chloride (Sigma-Aldrich) was first dissolved in deionized water to form a 100 mL solution at ≈1 M and left in room conditions to cool down to at least ≈26 °C. Then, the substrate was placed horizontally into an iron(III) chloride solution using a custom-designed mount. Meanwhile, the solution was placed in a water bath, into which a probe sonicator Vibra-Cell VCX 130 (Sonics) was inserted. Sonication proceeded continuously for 7.5 min at a 50% amplitude as the substrate was etched. After etching, the solution was increased in temperature to ≈30 °C. The sample was rinsed with deionized water and dried with nitrogen, followed by sonicating in deionized water for 10 min. The sample was dried with nitrogen again. For the microstructure etching for each substrate, a fresh 100 mL iron(III) chloride solution at ≈1 M was used.

### Fabrication of Nanostructures

Nanostructures were fabricated on aluminum using hot deionized water.^23,51,52^ First, >75 mL of deionized water was heated to ≈95 °C on a hot plate. The clean flat or microstructured substrates were then placed in hot water. After 10 min, deionized water at room temperature was added to quench the process. The samples were taken out and dried with nitrogen.

### pPFDA Coating

The samples were coated with pPFDA by using initiated chemical vapor deposition (iCVD). The substrates were first placed in oxygen plasma for 10 min at 0.6 mbar (Femto, Diener electronic). Next, they were transferred to a custom-made CVD chamber to form a coating of trichlorovinylsilane (Sigma-Aldrich). Using an iCVD system (iLab, GVD), these samples were coated with pPFDA at 100 mTorr, with a stage temperature of 40 °C and a filament temperature of 300 °C. *tert*-Butyl-peroxide (Sigma-Aldrich) was used as the initiator, and 1*H*,1*H*,2*H*,2*H*-perfluorodecyl acrylate (Sigma-Aldrich) was used as the monomer.

### FDTS and PFDTS Monolayer Application

Clean flat aluminum substrates were silanized by using FDTS and PFDTS via chemical vapor deposition. A vial was filled with 1 mL of hexane (Sigma-Aldrich) and 2 μL of silane (both from Sigma-Aldrich) in a nitrogen environment. This was subsequently opened and placed together with one substrate in a sealed beaker and then placed in an oven at 95 °C for 3 h. The substrate was finally cooled in ambient to room temperature.

### Surface Characterization

The surface morphology of H-pPFDA was acquired by means of a Hitachi SU8230 scanning electron microscope. A goniometer (OCA35, DataPhysics) was used to measure the contact angles on all of the surfaces. The images of the droplets were captured with a built-in camera and contact angles were measured using the software ImageJ.^64^

### Mean Droplet Departure Diameter and Number of Instantaneous Ejected Droplet Experiments

We estimated the mean droplet departure diameter of H-pPFDA and PTFE/CNF surfaces at different times during the durability test based on 20 random droplets for each time. The resolution limit was ≈25 μm per pixel, causing the smallest measurable droplets to have a diameter of ≈50 μm with consequent overestimation of the reported diameters.

Regarding the instantaneous number of ejected droplets, at each time during the durability test, we manually counted the number of droplets that left the surface by jumping using the ImageJ software.^64^ This was done for 3 random snapshots acquired via high-speed imaging for a given time (reported are the respective mean and standard deviation). For the case of JDWC, a portion of the droplet amount that left the surface could not be clearly detected because the droplets were either too small or out of focus. The reported data are therefore an underestimation of the JDWC mechanism due to inherent experimental limitations.

## Data Availability

All of the data used in the main manuscript and the SI to support the claims are available from the corresponding author upon reasonable request.

## Supporting Information Available

Optimization of the iCVD deposition process of pPFDA coating; condensation setup at high pressure; durability comparison of silanes monolayers and pPFDA coating; wettability comparison of the superhydrophobic microstructured surface before and after condensation exposure; condensation dynamics comparison at same subcooling between nanostructured and H-pPFDA surfaces; condensation setup and experiments at low pressure; structures morphology comparison before and after the durability test on H-pPFDA (PDF)
Condensation dynamics on FDTS-, PFDTS- and pPFDA-coated Al after ≈1h of steam exposure (MP4)
Durability of the H-pPFDA surface (the larger droplets visible on the top surface section are due to edge effects from the finite size of the test samples and do not represent any failure of the surface durability at these locations) (MP4)
