# Supplementary material for: Metal Surface Engineering for Extreme Sustenance of Jumping Droplet Condensation
Source: Langmuir. 2023 Dec 29;40(2):1257–65. doi: 10.1021/acs.langmuir.3c02713 (PMC10795172; doi:10.1021/acs.langmuir.3c02713)
Supplement: Supplementary file 1 — la3c02713_si_001.pdf [file la3c02713_si_001.pdf]

Supplementary Information

Metal surface engineering for extreme sustenance of jumping droplet condensation

Matteo Donati[1], Kartik Regulagadda[1], Cheuk Wing Edmond Lam[1,3], Athanasios Milionis[1], Chander Shekhar Sharma[2], and Dimos Poulikakos[1*]

[1]Laboratory of Thermodynamics in Emerging Technologies, Department of Mechanical and Process Engineering, ETH Zurich, Sonneggstrasse 3, 8092 Zurich, Switzerland.

[2]Thermofluidics Research Laboratory, Department of Mechanical Engineering, Indian Institute of Technology Ropar, Rupnagar 140001, Punjab, India.

[3]Present address: Massachusetts Institute of Technology, Cambridge, MA, 02139, United States.

[*]Corresponding Author

Prof. Dr. Dimos Poulikakos
Email: dpoulikakos@ethz.ch
Phone: +41 44 632 27 38
Fax: +41 44 632 11 76

Keywords: condensation, superhydrophobic surface, heat transfer

Table of Contents

S1 pPFDA Coating Thicknesses via iCVD: State-of-the-Art 5

S2 pPFDA Coating Thickness Measurement...6

S3 Condensation Setup at High Pressure...8

(a) Overall Experimental Setup and Procedure...8

(b) Heat Flux and Heat Transfer Coefficient Estimation 11

(c) Uncertainty Propagation 13

S4 Durability Comparison of Silanes Monolayers and pPFDA Coating 16

S5 Wettability Comparison of Superhydrophobic Microstructured Surface Before and After Condensation Exposure..17

S6 Condensation Dynamics Comparison at Same Subcooling Between Nanostructured and H-pPFDA Surfaces 18

S7 Condensation Setup and Experiments at Low Pressure 20

(a) Overall Experimental Setup and Procedure...20

(b) Condensation Heat Transfer Performance of H-pPFDA 21

S8 Structures Morphology Comparison Before and After Durability Test of H-pPFDA 23

S9 Condensation Heat Transfer Performance Comparison at High Pressure Between H-pPFDA and PTFE/CNF..24

S10 List of Supplementary Videos..25

S1 pPFDA Coating Thicknesses via iCVD: State-of-the-Art

Work	pPFDA Thickness
Coclite et al. (2012)[1]	≈ 300 nm
Paxson et al. (2014)[2]	≈ 40 nm
Khalil et al. (2019)[3]	≈ 40 nm
Tripathy et al.(2022)[4]	≈ 40 nm
Hoque et al.(2023)[5]	≈ 30 nm
This work	≈ 4.0 nm

Table S1 Overview on the state-of-the-art on pPFDA coating thicknesses achieved via iCVD.

S2 pPFDA Coating Thickness Measurement

The pPFDA coating thickness was measured by means of ellipsometry using a V-VASE ellipsometer (J.A. Woollam, USA). The thickness was estimated on reference silicon chips coated with the same iCVD process as the aluminum samples. After having defined material stack, a proprietary software fitted the measured data and estimates the pPFDA layer thickness. On each sample, measurements were taken at least at 2 locations, over wavelengths of 200 – 1600 nm, for at least 3 different angles. First, using a silicon chip cut from a pristine wafer, the native silicon dioxide thickness was determined to be ≈ 1.7 nm. This was done by defining a material stack consisting of silicon (525 µm) and SiO_2. As far as the pPFDA-coated samples are concerned, the pPFDA layer was added to the material stack as Cauchy material. This was done by using constants A: 1.3992, B: 0.0069215, C: -0.00024462, which were found to provide the best fit.[6] The thickness of the optimized pPFDA layer was determined to be ≈ 4.0 nm. An example for the data fitting at one location on this sample is displayed in Figure S1.

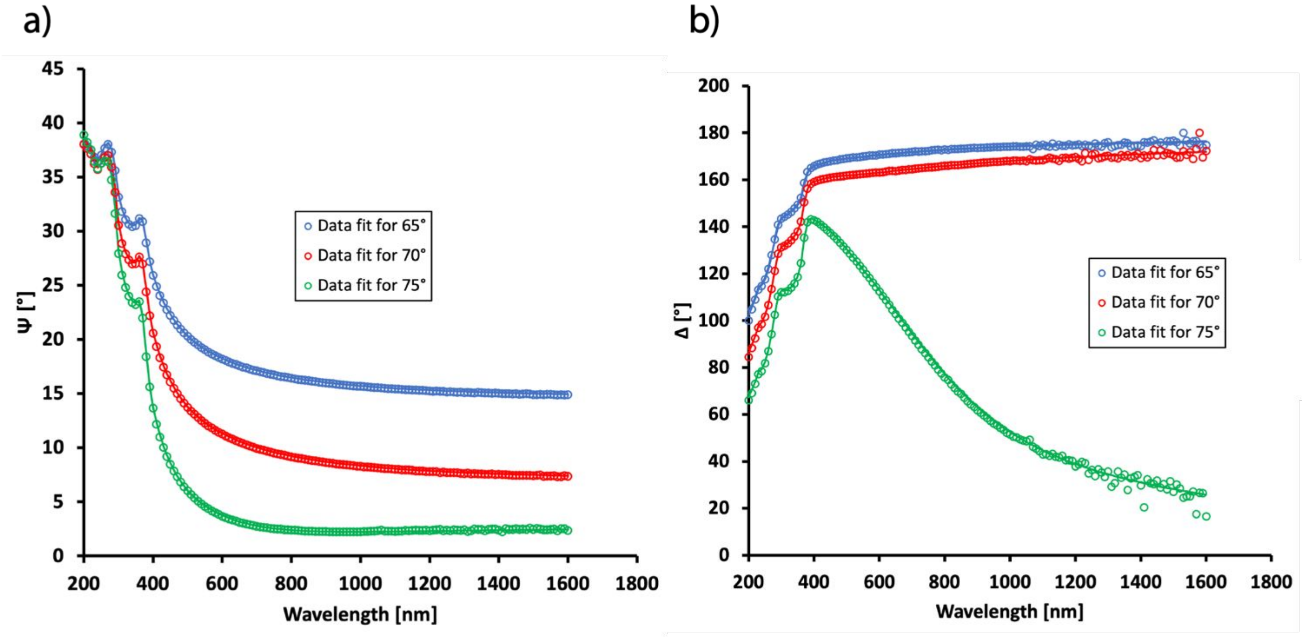

Figure S1 Raw ellipsometer data (circles) and the respective fit to the model (solid lines) at one location of a silicon wafer coated with pPFDA using the optimized deposition recipe. The different colors represent measurements at different angles. (a) Spectra of the amplitude component Ψ and (b) the phase difference Δ.

S3 Condensation Setup at High Pressure

(a) Overall Experimental Setup and Procedure

In this section, a description of the high-pressure condensation setup, as well as the experimental procedure, are reported. Figure S2 displays a schematic of the overall setup. The high-pressure flow chamber is integrated into a closed-loop system. This enables the performance of continuous experiments over prolonged periods. Steam is generated by means of a thermally-insulated boiler filled with deionized water. The steam velocity can be adjusted by controlling the heating power, which has a direct influence on the evaporation rate. To ensure steam free from liquid droplets flowing on the tested surface, a demister is placed before the test section. Additionally, a rope heater installed on the pipeline, is used to finely control the temperature of the steam entering the test section. Throughout all experiments, the steam temperature flowing on the test surface is maintained within 110.7 °C and 111.3 °C. The sample is mounted on a cooling copper block, which is cooled from the other side by means of a recirculating chiller (Chiller 1). A good thermal contact between sample and cooler is ensured by a thin (< 0.5 mm) indium foil. The thermal gradient in longitudinal direction along the copper cooler is measured by an array of 5 precisely positioned thermocouples. The copper cooler is properly insulated from all the sides by an air gap, such that one-dimensional heat conduction along the thermocouples array can be assumed. Figure S3 displays a cross section of the test chamber. A secondary condenser, connected to a second recirculating chiller (Chiller 2), is located after the test section, and is used to condense the residual steam. At this point the liquid water can flow back to the boiler by gravity. During the experiments, a needle valve is used to finely tune the loop pressure. For all the experiments the boiler pressure is maintained in the range 1.445 bar - 1.455 bar. Prior to each experiment, a vacuum pump is connected to the test chamber to minimize the presence of non-condensable gases. The pressure is

brought down to ≈ 15 mbar. After that, the entire loop is filled with deionized water by means of a second pump until the final pressure reaches ≈ 1.7 bar. After system stabilization, residues of non-condensable gases migrate to the setup top and can be removed using a valve located at that position. After that, water is pumped again, and the aforementioned steps are repeated until no more non-condensable gases are left in the setup. During this procedure, the setup pressure is always kept > 1.1 bar to avoid inward leakages of air. At this point, the boiler is heated up until steam is generated. During this part, the pressure of the setup is reduced by removing air from a pressure vessel, which was previously filled with pressurized air. After steam flow generation, this can be stabilized by means of a needle valve. We report heat transfer measurements at steam velocities of 3 and 9 m/s, which represent the minimum and maximum attainable values. By varying the coolant temperature of Chiller 1, the subcooling can be changed. All the sensors are connected to a data acquisition system.

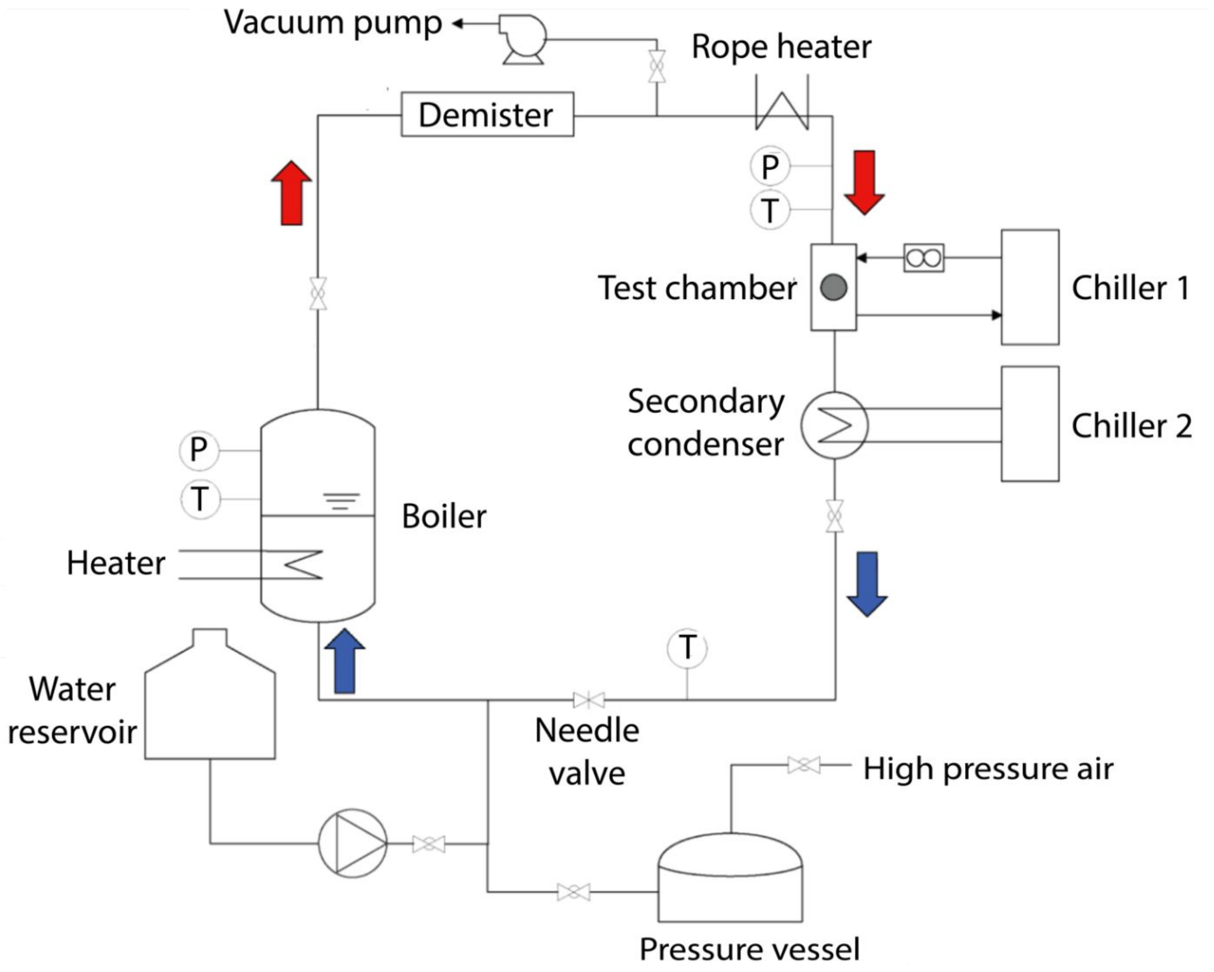

Figure S2 Schematic of the high-pressure experimental setup. Adapted from *Donati et al.*[7].

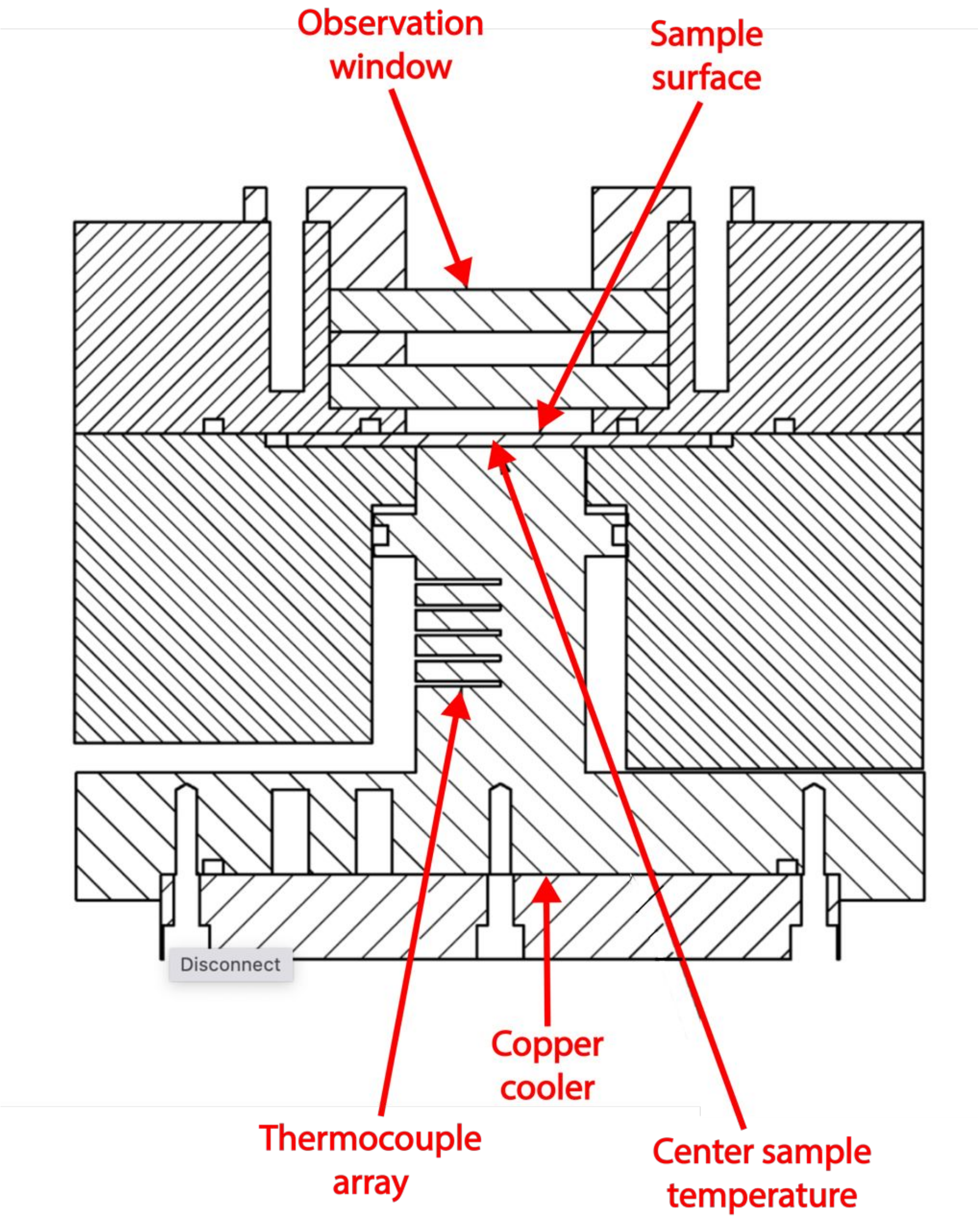

Figure S3 Cross section of the chamber of the high-pressure condensation setup. Adapted from *Donati et al.*[7].

(b) Heat Flux and Heat Transfer Coefficient Estimation

The heat flux q'' flowing through the surface is computed by means of a least-square linear fitting of the temperature gradient along the thermocouple array embedded in the copper cooler. This is done by assuming 1-D heat conduction:

$$q'' = \lambda \frac{dT}{dx}$$

λ is the thermal conductivity of the cooler and $\frac{dT}{dx}$ represents the thermal gradient. Since the cooler cross-sectional area and the condensing surface are equal, the heat flux flowing through them, is the same. The steam temperature T_{steam} is directly measured at the test section inlet by means of a thermocouple. A second thermocouple is inserted in a microhole drilled in the sample from the side. This is used to measure the temperature below the center of the condensing surface (T_b) at a depth $t_{substrate}$. The sample surface temperature T_s is estimated by means of T_b and q'' as follows:

$$T_s = T_b + q'' \frac{t_{substrate}}{k_{substrate}}$$

$t_{substrate}$ represents the thickness of the substrate between surface and the location of the thermocouple measuring T_b, while $k_{substrate}$ is the substrate thermal conductivity. T_s is then used to estimate the HTC h as follows:

$$h = \frac{q''}{\Delta T} = \frac{q''}{T_{steam} - T_s}$$

$\Delta T = T_{steam} - T_s$ represents the subcooling. The heat transfer values presented here rely on steady state measurements, where a set of measured quantities must satisfy a specific condition, consisting of the maximum standard deviation of 120 readings acquired over a 2-minute period. The maximum standard deviation is chosen separately for each quantity. Once steady state is confirmed, the mean values recorded over 2 minutes are adopted as steady state measurements. The uncertainties are calculated using the error propagation method, by considering the standard

deviation of the aforementioned 120 measurements and uncertainty of each sensor as described in the next section.

(c) Uncertainty Propagation

In this section, similarly to *Donati et al.*[7], the procedure for subcooling, heat flux and HTC uncertainties computation, is described.

The uncertainty in steam temperature $\delta_{T_{steam}}$ is given by:

$$\delta_{T_{steam}} = \sqrt{\sigma_{T_{steam}}^2 + \delta_{T_{steam,\,cal}}^2}$$

$\sigma_{T_{steam}}$ is the standard deviation of T_{steam} calculated based on 120 readings, while $\delta_{T_{steam,cal}}$ is the uncertainty from the calibration of the thermocouple measuring T_{steam}.

Similarly, the sample central temperature uncertainty δ_{T_b} is provided by:

$$\delta_{T_b} = \sqrt{\sigma_{T_b}^2 + \delta_{T_b}^2}$$

where σ_{T_b} is the standard deviation of T_b calculated based on 120 readings, while δ_{T_b} is the uncertainty from the calibration of the thermocouple measuring T_b.

The uncertainty in the estimated surface temperature δ_{T_s} is then propagated:

$$\delta_{T_s} = \sqrt{\delta_{T_b}^2 + \delta_{q''}^2 \left(\frac{t_{substrate}}{k_{substrate}}\right)^2}$$

where $\delta_{q''}$ is the heat flux uncertainty.

The uncertainty in subcooling is then computed as follows:

$$\sigma_{\Delta T} = \sqrt{\delta_{T_{steam}}^2 + \delta_{T_s}^2}$$

The uncertainty in heat flux corresponds to the uncertainty in the determination of the temperature gradient slope along the cooler. The linear fit uncertainty δ_{lin_fit} is calculated as follows:

$$\delta_{lin\_fit} = \delta_{TC}\sqrt{\frac{N_{TC}}{N_{TC}\sum x_{TC}^2 - \left(\sum x_{TC}\right)^2}}$$

δ_{TC} is the uncertainty in the temperature measurement of a thermocouple, $N_{TC} = 5$ corresponds to the total number of thermocouples along the cooler and x_{TC} is the location of the thermocouples along the array.

δ_{TC} is estimated by:

$$\delta_{TC} = \sqrt{\sigma_{T,max}^2 + \delta_{cal}^2 + \delta_{eval,\ lin\_fit}^2}$$

$\sigma_{T,max}$ corresponds to the maximum 120-reading standard deviation from the cooler thermocouples (thus maximum of 5 standard deviation values) during the stady state measurement, δ_{cal} is the uncertainty from the thermocouples calibration and δ_{eval,lin_fit} is the uncertainty in the linear fit evaluation. δ_{eval,lin_fit} is computed by:

$$\delta_{eval,lin_{fit}} = \sqrt{\frac{1}{N_{TC} - 2}\sum_{i=1}^{N_{TC}}(T_i - B - Ax_i)^2}$$

T_i and x_i are the temperature measured by the ith thermocouple and its position along the array, respectively.

The uncertainty in heat flux corresponds to the uncertainty in the linear fit multiplied by the cooler thermal conductivity k:

$$\delta_{q''} = k\delta_{lin\_fit}$$

The HTC h is calculated as the ratio between the heat flux and the subcooling. Its uncertainty δ_h is propagated as follows:

$$\delta_h = \sqrt{\left[\frac{1}{(\Delta T)}\delta_{q''}\right]^2 + \left[\frac{-q''}{(\Delta T)^2}\delta_{T_{steam}}\right]^2 + \left[\frac{q''}{(\Delta T)^2}\delta_{T_s}\right]^2}$$

S4 Durability Comparison of Silanes Monolayers and pPFDA Coating

As evidenced by the experimental results, although both, silanes and pPFDA, have the same hydrolytically unstable (Si-O) chemical bonds with the substrate,[8,9] the pPFDA coating shows a significantly enhanced durability under condensation exposure compared to FDTS and PFDTS. The silane monolayers used in this work consists of an array of vertically oriented molecules with hydrophobic chains. Because of the inherent presence of defects, water vapor can easily reach the Si-O bonds and break them, causing fast coating removal.[10,11] On the other hand, the pPFDA coating is present in the form of highly densely packed hydrophobic chains.[1] We believe that this inherently leads to a significant defect protection to the Si-O bonds formed during the silanization step. Thus, it is harder for the water molecules to break the Si-O bonds and subsequently remove the pPFDA coating.

S5 Wettability Comparison of Superhydrophobic Microstructured Surface Before and After Condensation Exposure

	ACA	CAH
Before	160.5° ± 4.5°	19.4° ± 2°
After	161.1 ± 5.1°	21.2° ± 3°

Table S2 Wettability of superhydrophobic microstructured aluminum coated with pPFDA before and after condensation heat transfer experiment.

S6 Condensation Dynamics Comparison at Same Subcooling Between Nanostructured and H-pPFDA Surfaces

A comparison between the condensation dynamics on the superhydrophobic nanostructured and hierarchically-structured aluminum (both coated with pPFDA) at the same subcooling of ≈ 3.6 K reveal a significant difference in condensate removal mechanism. As far as the surface with nanostructures only is concerned, this shows a mix between DWC and JDWC, caused by partial surface flooding. On the other hand, the H-pPFDA displays practically exclusively JDWC, proving that the addition of microstructures has a significant positive impact in condensate removal. This is reflected in the HTC. In fact, in the case of hierarchical texture the HTC is ≈ 1.5 × higher compared to the nanostructured surface. Figure S4 shows a snapshot during condensation on the two aforementioned surfaces at the same subcooling. A direct condensation mode comparison between the two surfaces can be seen in Supplementary Video V1.

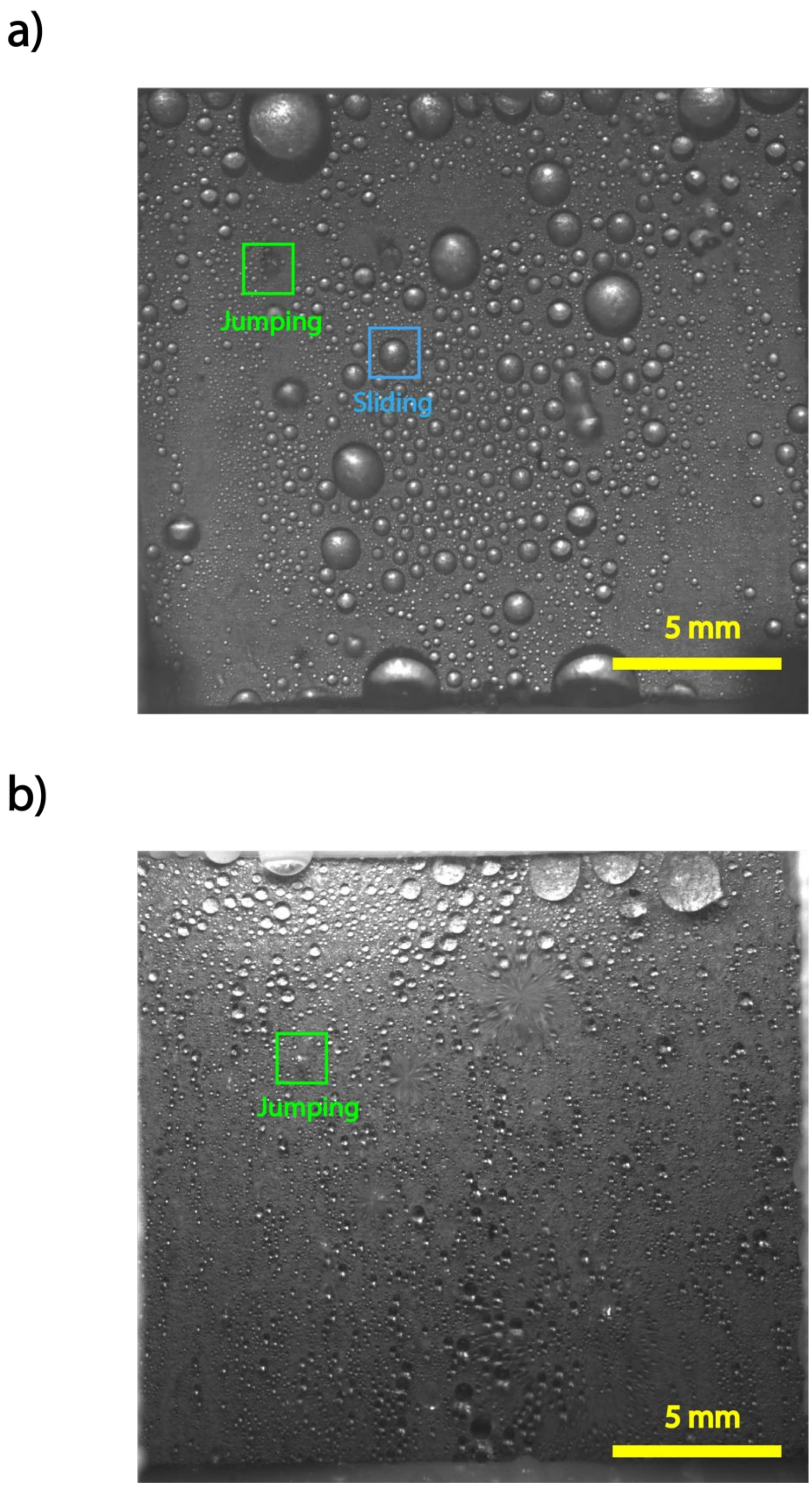

Figure S4 Condensation mode at same subcooling on superhydrophobic pPFDA-coated (a) nanostructured and (b) hierarchically-structured aluminum. The green and blue squares highlights examples of jumping and sliding droplets respectively.

S7 Condensation Setup and Experiments at Low Pressure

(a) Overall Experimental Setup and Procedure

The samples were put to test under industrially relevant condensation conditions to examine their heat transfer performance. We used a custom-built chamber and follow similar experimental procedures as described in our previous work.[12] In the following, a summary is provided.

We exposed our samples to 30 mbar saturated steam for condensation, corresponding to a saturation temperature of 24 ˚C. In our chamber, the samples are placed vertically, and horizontal steam flow is continuously maintained with a constant source (boiler of constant pressure) and a constant sink (pump). The resulting mean flow speed over the sample is ≈ 4.0 – 5.2 m s^{-1} in a channel of height 10 mm. The flow speed depends on the heat flux, as to maintain the chamber pressure a higher condensation rate requires a higher steam inflow rate.[12]

The temperatures of the condensing surface and the steam are each measured from the mean of two resistance temperature detectors (RTDs). The steam pressure is measured with a capacitance gauge. The sample is placed on a heat sink cooled with a recirculating chiller. In the heat sink, a linear array of 7 RTDs measure the heat flow through the heat sink, and thus the sample, using the Fourier's Law. Heat flux through the sample is obtained by dividing the heat flow with the exposed area for condensation. The subcooling is defined as the difference between the steam temperature and the condensing surface temperature. Dividing the heat flux with subcooling, the heat transfer coefficient is computed.

Before each experiment, the chamber is evacuated to < 0.01 mbar overnight to remove remaining condensate from previous experiments and other volatile contaminants. The sample is then mounted, and the chamber is evacuated to < 0.01 mbar again. From this point, the pump

continuously operates until the end of the experiment. We first vigorously boil deionized water for at least 30 min to reduce dissolved gases. Meanwhile, the heat sink is set to a temperature of 25 ˚C. To begin the experiment, steam from the boiler is introduced through metering and regulating valves into the chamber to achieve the target pressure of 30 mbar. As the target pressure is reached, the temperature of the heat sink is reduced to 20 ˚C. A subcooling is established and condensation begins. As the system reaches steady state, we measure the data for 1 min at 2 Hz without intervention. The temporal mean is taken as the data point at this heat sink temperature, and error bars are computed as described in our previous work.[12]

After that, we reduce the heat sink temperature at 5 ˚C intervals to -10 ˚C. At each heat sink temperature, we repeat the steady state measurement, obtaining 7 measurements in total after the last at -10 ˚C. At the end of the experiment, the heat sink is set to heat up again. Steam flow is shut off and the chamber is vented when the heat sink reaches a temperature above freezing. The sample is removed and blown dry with nitrogen.

(b) Condensation Heat Transfer Performance of H-pPFDA

We evaluate the condensation heat transfer performance of H-pPFDA in the experimental setup. Figure S5 displays heat transfer coefficient and heat flux vs. subcooling. H-pPFDA shows a ≈ 2.9 × higher HTC compared to FWC.

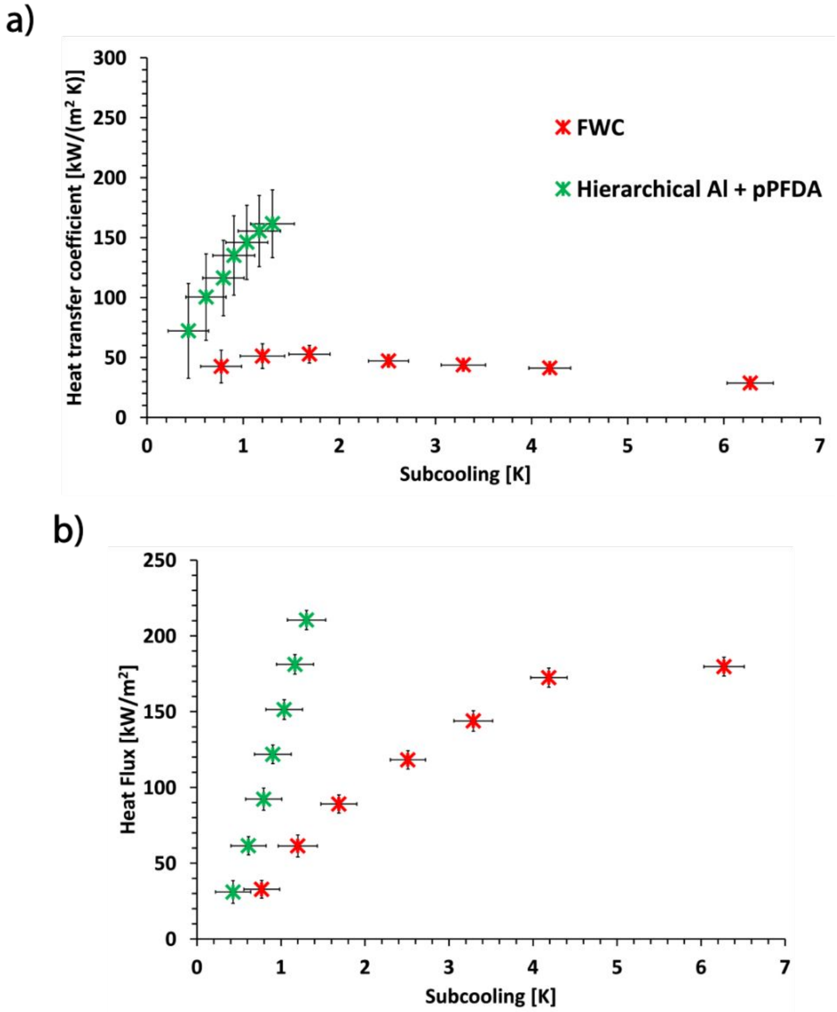

Figure S5 (a) Heat transfer coefficient and (b) heat flux vs. subcooling for H-pPFDA surface.

S8 Structures Morphology Comparison Before and After Durability Test of H-pPFDA

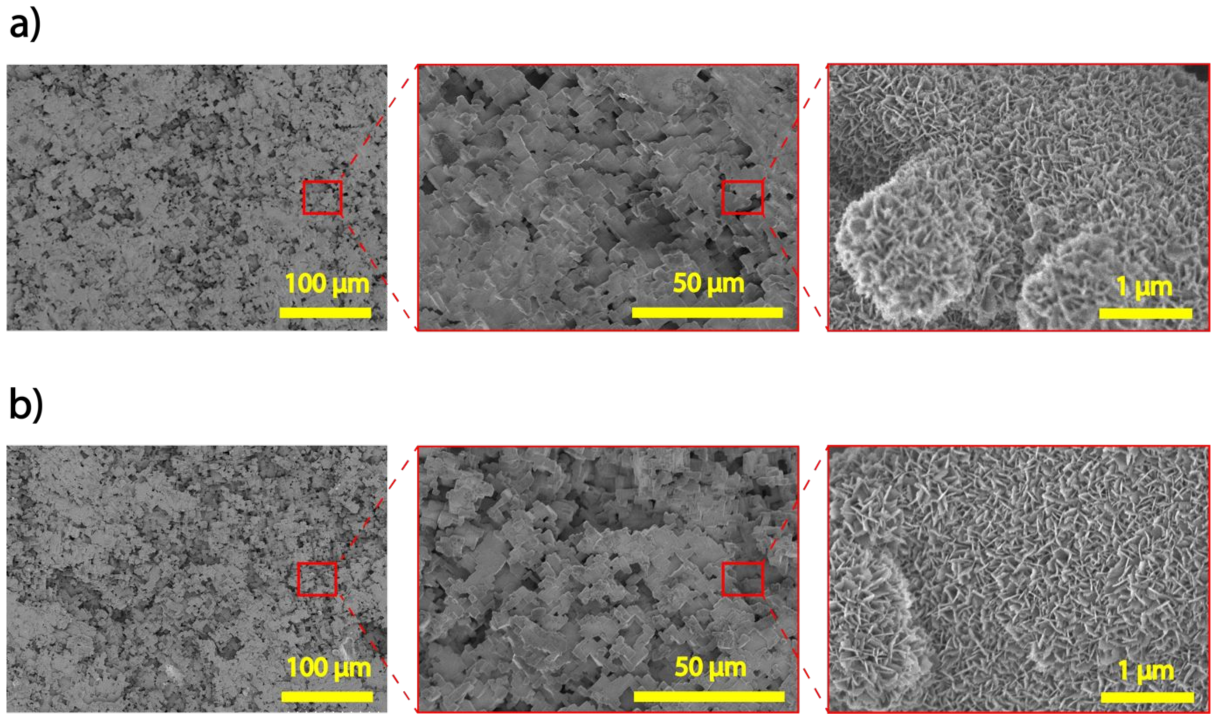

Figure S6 Surface morphology comparison on H-pPFDA surface (a) before and (b) after the durability test.

S9 Condensation Heat Transfer Performance Comparison at High Pressure Between H-pPFDA and PTFE/CNF

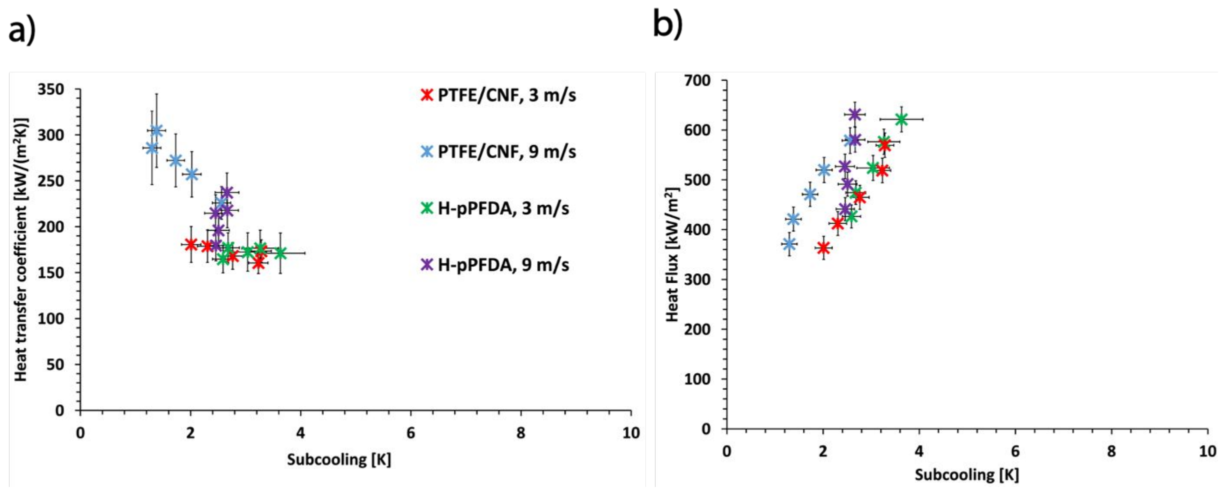

Figure S7 Condensation heat transfer performance of PTFE/CNF and H-pPFDA surfaces under steam shear flow at high pressure at 3 and 9 m/s. (a) Heat transfer coefficients and (b) heat fluxes. Data of PTFE/CNF adopted from *Donati et al.*[7]

S10 List of Supplementary Videos

V1. Condensation dynamics on FDTS-, PFDTS- and pPFDA-coated Al after ≈ 1h of steam exposure. (MP4)

V2. Durability of the H-pPFDA surface. The larger droplets visible on the top surface section are due to edge effects from the finite size of the test samples and do not represent any failure of the surface durability at these locations. (MP4)

References

(1) Perrotta, A.; Christian, P.; Jones, A. O. F.; Muralter, F.; Coclite, A. M. Growth Regimes of Poly(Perfluorodecyl Acrylate) Thin Films by Initiated Chemical Vapor Deposition. *Macromolecules* **2018**, *51* (15), 5694–5703.

(2) Paxson, A. T.; Yagüe, J. L.; Gleason, K. K.; Varanasi, K. K. Stable Dropwise Condensation for Enhancing Heat Transfer via the Initiated Chemical Vapor Deposition (ICVD) of Grafted Polymer Films. *Adv. Mater.* **2014**, *26* (3), 418–423.

(3) Khalil, K.; Soto, D.; Farnham, T.; Paxson, A.; Katmis, A. U.; Gleason, K.; Varanasi, K. K. Grafted Nanofilms Promote Dropwise Condensation of Low-Surface-Tension Fluids for High-Performance Heat Exchangers. *Joule* **2019**, *3* (5), 1377–1388.

(4) Tripathy, A.; Regulagadda, K.; Wing, C.; Lam, E.; Donati, M. A.; Milionis, A.; Sharma, C. S.; Mitridis, E.; Schutzius, T. M.; Poulikakos, D. Ultrathin Durable Organic Hydrophobic Coatings Enhancing Dropwise Condensation Heat Transfer. *Langmuir* **2022**, *38* (37), 11296–11303.

(5) Hoque, M. J.; Li, L.; Ma, J.; Cha, H.; Sett, S.; Yan, X.; Rabbi, K. F.; Ho, J. Y.; Khodakarami, S.; Suwala, J.; Yang, W.; Mohammadmoradi, O.; Ince, G. O.; Miljkovic, N. Ultra-Resilient Multi-Layer Fluorinated Diamond like Carbon Hydrophobic Surfaces. *Nat. Commun.* **2023**, 14, 4902.

(6) Cheuk, W. E. L.; Regulagadda, K.; Donati, M.; Tripathy, A.; Chandra Pal, G.; Shekhar Sharma, C.; Milionis, A.; Poulikakos, D. Condensate Droplet Roaming on Nanostructured Superhydrophobic Surfaces. *arXiv,* ***2023****. preprint arXiv:2310.11382*.

(7) Donati, M.; Lam, C. W. E.; Milionis, A.; Sharma, C. S.; Tripathy, A.; Zendeli, A.; Poulikakos, D. Sprayable Thin and Robust Carbon Nanofiber Composite Coating for Extreme Jumping Dropwise Condensation Performance. *Adv. Mater. Interfaces* **2021**, *8* (1),

(8) Arkles, B. *Silane Coupling Agents: Connecting Across Boundaries*; 2014. www.gelest.com.

(9) Dow Corning. *The Concept of Coupling with Organofunctional Silanes*. https://krayden.com/pdf/xia_silane_chemistry.pdf (accessed 2023-10-27).

(10) Zhizhchenko, A. Y.; Shabalina, A. V.; Aljulaih, A. A.; Gurbatov, S. O.; Kuchmizhak, A. A.; Iwamori, S.; Kulinich, S. A. Stability of Octadecyltrimethoxysilane-Based Coatings on Aluminum Alloy Surface. *Materials* **2022**, *15* (5).

(11) Wang, R.; Jakhar, K.; Ahmed, S.; Antao, D. S. Elucidating the Mechanism of Condensation-Mediated Degradation of Organofunctional Silane Self-Assembled Monolayer Coatings. *ACS Appl. Mater. Interfaces* **2021**, *13* (29), 34923–34934.

(12) Tripathy, A.; Lam, C. W. E.; Davila, D.; Donati, M.; Milionis, A.; Sharma, C. S.; Poulikakos, D. Ultrathin Lubricant-Infused Vertical Graphene Nanoscaffolds for High-Performance Dropwise Condensation. *ACS Nano* **2021**, *15* (9), 14305–14315.
